# Comparison of Inhibitory Effects of Cinnamic Acid, β-Cyclodextrin, L-Cysteine, and Ascorbic Acid on Soluble and Membrane-Bound Polyphenol Oxidase in Peach Fruit

**DOI:** 10.3390/foods12010167

**Published:** 2022-12-29

**Authors:** Shuang Jia, Shu Jiang, Yi Chen, Yingying Wei, Xingfeng Shao

**Affiliations:** Zhejiang-Malaysia Joint Research Laboratory for Agricultural Product Processing and Nutrition, College of Food and Pharmaceutical Sciences, Ningbo University, Ningbo 315800, China

**Keywords:** polyphenol oxidase (PPO), membrane-bound, inhibition, direct effect, peach fruit

## Abstract

There has been considerable interest in controlling polyphenol oxidase (PPO) activity to prevent enzymatic browning in foods. However, studies on inhibitions of different forms of PPO are very limited. Thus, this study focuses on the effects of cinnamic acid, β-cyclodextrin, L-cysteine, and ascorbic acid on soluble PPO (sPPO) and membrane-bound PPO (mPPO) in peach fruit. The activity of partially purified sPPO was 3.17 times higher than that of mPPO. However, mPPO was shown to be more stable than sPPO in the presence of inhibitors with different concentrations (i.e., 1, 3, 5 mM); activation of mPPO was found by 5 mM L-cysteine. Both sPPO and mPPO inhibitions were PPO substrate-dependent. Ascorbic acid showed the highest inhibitory effect on both sPPO and mPPO with all studied inhibitors and substrates. The inhibition of 1 mM ascorbic acid on sPPO and mPPO reached 95.42 ± 0.07% and 65.60 ± 1.16%, respectively. β-Cyclodextrin had a direct inhibitory effect only on sPPO, while the other three inhibitors had direct effects on both sPPO and mPPO. Cinnamic acid exhibited a non-competitive inhibition on sPPO and mPPO, with L-cysteine showing the same, though on sPPO. The inhibition of studied inhibitors on sPPO and mPPO is highly related to the substrate environment, type, and concentration of inhibitors. This study provides a basis for the further prevention of peach fruit browning from the perspective of different enzyme forms.

## 1. Introduction

Peach (*Prunus persica* L.) fruit is delicious and rich in various nutrients (e.g., dietary fiber, organic acids, vitamins, phenolic compounds, and other bioactive substances) [1]. For these reasons peach fruit is deeply loved by the public. However, due to the thin skin and the soft flesh of the peach fruit, it is extremely susceptible to mechanical damage during postharvest handling, storage and processing, resulting in enzymatic browning [2]. Also, as peach is a climacteric fruit, low-temperature (2–8 °C) storage of peach fruit for long periods of time leads to severe internal browning due to cell membrane damage under chilling conditions [3].

Undesired enzymatic browning is a great concern in the food industry due to its negative effects on food quality, resulting in large amounts of food waste. It has been reported that more than 50% losses in the fruits and vegetables are directly or indirectly caused by enzymatic browning [4]. This browning is initiated by polyphenol oxidase (PPO, EC 1.14.18.1)-catalyzed oxidations of phenolic compounds under aerobic conditions. PPO is a common copper-containing enzyme and the copper atom in its active center plays a crucial role in catalyzing its activity [5]. PPO commonly exists in plant chloroplast thylakoids in two forms: a soluble form (sPPO) remaining in the thylakoid lumen and a membrane-bound form (mPPO) bound to the thylakoid membrane [5]. Relevant studies have shown that sPPO may be the result of the spontaneous release of mPPO through a series of biochemical reactions during the growth and development of fruits from ripening to senescence [6]. The proportional relationship between sPPO and mPPO is highly influenced by plant species, growth and storage conditions, and processing methods [7,8,9]. Studies on PPO mainly focus on the extraction, isolation, and purification of total PPO and their basic enzymatic properties in various fruit and vegetables, such as loquat [10], jackfruit [11], purple sweet potato [12] and water yam [13]. Although mPPO in some fruits and vegetables (e.g., Fuji apple, snake fruit, “Xushu 22” sweet potato skin) has become a research interest in recent years [14,15,16], different forms of PPO (i.e., sPPO, mPPO) in peach fruit are rarely studied.

There remains considerable interest in developing effective methods to control browning in foods. Chemical methods for browning inhibition are often widely studied because they can effectively control browning and are easy to use in foods. Chemical inhibitors that have been studied include carboxylic acids, ascorbic acid and its derivatives, sulfur-containing compounds, phenolic acids and cyclodextrins [17,18,19,20].

Among these, cinnamic acid, β-cyclodextrin (βCyD), L-cysteine (L-Cys), and ascorbic acid (AA) are often used as representative anti-browning agents and have been shown to effectively inhibit the PPO activity and browning in various fruit and vegetables (e.g., pear, African bush mango fruit peel, mung bean sprout, fresh-cut potatoes) [21,22,23,24,25]. Although there are many studies focusing on the inhibitory effect of different chemical inhibitors on PPO, such an effect is mainly investigated on total PPO. However, the effect of these common food-grade anti-browning agents on different forms of PPO is unclear and studies on inhibition of various inhibitors on sPPO and mPPO are very limited. Most of the studies only focus on screening sPPO or mPPO inhibitors for the purpose of determining the better inhibitors, but the specific inhibition mechanisms on sPPO or mPPO were not comprehensively studied [7]. As sPPO and mPPO may behave differently in the presence of different inhibitors, the effects of inhibitors on the two different enzyme forms need to be determined for better understanding of browning inhibition in foods. 

The aim of the present study was to determine the effects of different inhibitors on sPPO and mPPO in peach fruit. Four common and relatively safe chemical anti-browning agents, including cinnamic acid, βCyD, L-Cys, and AA, were selected as representative inhibitors, with the endogenous substrate, chlorogenic acid (CA), being used as the study PPO substrate. This contributes to a further understanding of PPO inhibition with respect to two different PPO forms; such a study can provide a theoretical basis for preventing enzymatic browning in peach fruit and further prolonging the shelf life of its related products.

## 2. Materials and Methods

### 2.1. Materials

Phenylmethylsulfonyl fluoride (PMSF), chlorogenic acid (CA), bovine serum albumin, β-cyclodextrin (βCyD), and ascorbic acid (AA) were obtained from Shanghai Yuanye Bio-Technology Co., Ltd. (Shanghai, China). Benzamidine hydrochloride, L-cysteine (L-Cys), and cinnamic acid were purchased from Shanghai Macklin Biochemical Technology Co., Ltd. (Shanghai, China). Triton X-114 and Comas Bright Blue G-250 were purchased from Solarbio (Beijing, China). Ammonium sulfate, sodium dihydrophosphate, sodium hydroxide, phosphoric acid, catechol, and caffeic acid were purchased from China Pharmaceutical Group Chemical Reagents Co., Ltd. (Shanghai, China).

Peaches (*Prunus persica* L. cv. HuJingMiLu) were harvested from the Peach Research Institute in Fenghua, Zhejiang Province, China. All peaches were at the same commercial maturity and from the same orchard. Peach fruit with uniform size and without obvious damages, diseases, or other defects were selected for experiments. On the harvest day, fresh peach fruit was peeled, cut into small pieces, and immediately frozen in liquid nitrogen. Frozen samples were then ground and stored at −80 °C until use.

### 2.2. Extraction of sPPO and mPPO

sPPO and mPPO were extracted according to the method proposed by Cabanes et al. [26], with slight modifications. Frozen peach fruit powder (100 g) was homogenized in 200 mL of cold 100 mM sodium phosphate buffer (pH 7.3) containing 1 mM benzamidine hydrochloride, 1 mM PMSF, and 10 mM AA for 60 s. PMSF was added immediately before use. The obtained homogenate was then centrifuged at 4000 g at 4 °C for 10 min; the supernatant was collected, and the precipitate containing mPPO was retained for the subsequent extraction of mPPO. The supernatant was centrifuged at 14,000 g for 30 min at 4 °C; the resulting supernatant contained the crude sPPO, and was kept at 4 °C for further purification. 

To further extract mPPO, an aliquot of 40 mL of 10 mM phosphate buffer (pH 7.3) containing 6% (*w*/*v*) TritonX-114 was slowly added to dissolve the precipitate that contained mPPO; the suspension was continuously stirred for 5 min and incubated at 4 °C for 1 h. This suspension was then kept at 37 °C for 15 min and the temperature-induced phases occurred; this was further centrifuged at 10,000 g at 25 °C for 15 min to reach better separation of the different phases. The upper aqueous phase was taken as the crude mPPO and this was stored at 4 °C for further purification.

### 2.3. Partial Purification of sPPO and mPPO

The crude extracts of sPPO and mPPO were precipitated by 25–80% (*w*/*v*) ammonium sulfate and the mixtures were centrifuged at 14,000 g for 30 min at 4 °C. After centrifugation, the collected precipitate was resuspended in 10 mM sodium phosphate buffer (pH 7.3), mixed well, and centrifuged at 4 °C at 14,000 g for 10 min to obtain the supernatant. The resulting supernatant was then dialyzed in 10 mM sodium phosphate buffer (pH 7.3) using a dialysis bag (8–14 kDa) with 3 changes of buffer within 24 h at 4 °C. The dialyzed solution is referred to as the partially purified sPPO and mPPO, respectively; these were stored at −40 °C for subsequent experiments.

### 2.4. Quantification of PPO Activity

The determination of PPO activity was slightly modified according to the method of Guo et al. [27]. A typical assay system contained 3 mM CA in 50 mM sodium phosphate buffer (pH 7.0) and prepared PPO. Reactions were initiated by adding 0.2 mL of mPPO or sPPO to 2 mL of CA solution at ambient temperature (~25 °C). The increase in absorbance due to product accumulation was measured at 420 nm spectrophotometrically, and the initial velocities were calculated based on the initial linear part of the reaction curves. One unit (U) of PPO activity was defined as a change in absorbance of 0.01 per minute.

### 2.5. Determination of Protein Content

Protein content of sPPO and mPPO was estimated using the Bradford method [28], with bovine serum albumin as the reference standard.

### 2.6. Effects of Inhibitors on sPPO and mPPO Activities

The four inhibitors evaluated in the present study were cinnamic acid, βCyD, L-Cys, and AA; final concentrations of each inhibitor were 1, 3, and 5 mM. For inhibition studies, 1 mL of CA substrate solution (final concentration of 3 mM) was mixed with 1 mL of the inhibitor solution. The PPO reactions were initiated immediately by the addition of 0.2 mL of sPPO or mPPO solution to 2 mL CA and inhibitor mixture, at ambient temperature (~25 °C). PPO activities were determined according to the method described in the Section 2.4. The percentage of PPO inhibition was calculated using the following equation [29]:

PPO Inhibition (%) = [(A_o_ − A_i_)/A_o_] × 100%, (1)

where A_o_ represents the enzyme activity without inhibitors, and A_i_ represents the enzyme activity with inhibitors.

### 2.7. Effects of Inhibitors on sPPO- and mPPO-Catalyzed Reactions with Different Substrates

The different substrates used in this experiment included CA, caffeic acid, and catechol, with final concentrations of 3, 10, and 10 mM in PPO reaction systems, respectively. Inhibitors used were as described above in Section 2.6, with a final concentration of 3 mM for each inhibitor. All substrates and inhibitors solutions were prepared using 50 mM sodium phosphate buffer solution (pH 7). As described above, 1 mL of substrate solution was mixed with 1 mL of the inhibitor solution, and 0.2 mL of sPPO or mPPO solution was then added to 2 mL of the mixture containing substrate and inhibitor to initiate the PPO reaction (~25 °C). PPO activity and percentage of inhibition were determined as described above.

### 2.8. Direct Effects of Inhibitors on sPPO and mPPO Activities

Determination of the inhibitors’ direct effects on PPO activity was based on preincubation of inhibitors with PPO for different periods of time prior to measure enzyme activity using CA as substrate. The inhibitor (i.e., cinnamic acid, βCyD, L-Cys, AA) solution (final mixture concentration of 3 mM) was mixed with sPPO or mPPO solution. The mixtures were then incubated for different periods of time (30, 60, 90, 120, and 150 min) at 4 °C before being tested for PPO activity. PPO reactions were then initiated by adding 0.2 mL of the incubation mixture to 2 mL of CA solution (final concentration of 3 mM CA). Initial velocities were measured as specified previously. Control experiments were treated identically but without inhibitors.

### 2.9. Comparison of Inhibition Types of sPPO and mPPO

Inhibitors used were as described above in the Section 2.7. In fixed-inhibitor assays, reactions were initiated by adding 0.2 mL of sPPO or mPPO solution to 2 mL CA solutions (ranging from 0.5–10 mM CA in 50 mM sodium phosphate buffer, pH 7) with or without inhibitors. Enzyme activities were determined as described above. Kinetic parameters (K_m_, V_max_) were estimated based on the Michaelis equation by non-linear least square fitting [20].

### 2.10. Statistical Analysis

All experimental data were reported as means ± standard deviation (SD) of triplicate experiments. Statistical differences between different groups were determined by analysis of variance (ANOVA) and paired samples t-test using the software SPSS 25.0, and means were compared using Duncan’s multiple range test (*p* < 0.05).

## 3. Results and Discussion

### 3.1. Comparison of sPPO and mPPO Activities

Initial experiments focused on comparing the activities of two different forms of sPPO and mPPO, including the activities of crude PPO extract and partially purified PPO. CA was used as the main PPO substrate in the present study, as CA is the predominant phenolic substrate that is endogenous to peach fruit [1]. As shown in Table 1, the specific activities of sPPO and mPPO after partial purification were 95 U/mg and 30 U/mg, respectively. Compared with the crude extract, the specific activities of sPPO and mPPO after partial purification were increased by 1.3 and 1.2 times; sPPO activity was 3.17-fold higher than that of mPPO, and the result was significant, according to the t-test (*p* < 0.05). This indicates that sPPO is highly likely to be the dominant enzyme form that participates in enzymatic browning reactions in ‘HuJingMiLu’ peach fruit. Compared with sPPO, mPPO may not be the main concern to control the browning of ‘HuJingMiLu’ peach fruit. However, this finding is inconsistent with previous studies on peach PPO. In ‘Lijiang’ snow peach, the specific activity of mPPO was 6.4-fold higher than that of sPPO, so mPPO was concluded to be the main form of PPO enzymes and the main factor that causes the browning [30]. The activity of mPPO in snake fruit was also higher than that of sPPO. This is probably because mPPO in snake fruit can be more easily activated, resulting in enzymatic browning [14]. The differences show that the dominant enzyme form for PPO that plays a major role in enzymatic browning can probably be dependent on fruit varieties and species.

### 3.2. Effects of Inhibitors on sPPO and mPPO Activities

**Figure 1:**
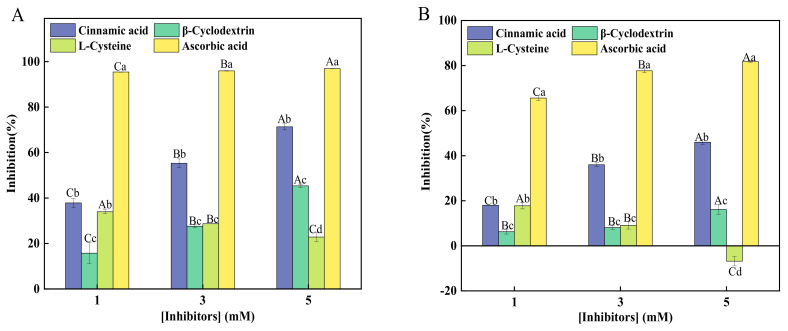
Effects of different inhibitors on the activities of sPPO (**A**) and mPPO (**B**) in ‘HuJingMiLu’ peach fruit. Inhibitor concentrations studied in this experiment included 1, 3, and 5 mM. Enzyme activities were determined using CA as the substrate. Different lowercase letters indicate that the inhibitory effects of different inhibitors at the same concentration are significantly different; different capital letters indicate that the inhibitory effects of the same inhibitor at different concentrations have significant differences (*p* < 0.05).

The effects of four different types of inhibitors on the activities of sPPO and mPPO were compared (Figure 1). As the concentration of these four inhibitors increased, the percent inhibition of cinnamic acid, βCyD, and AA on sPPO and mPPO activities significantly increased. AA shows the highest inhibition on both sPPO and mPPO activities, followed by cinnamic acid at all studied concentrations. At the lowest concentration (1 mM), the percent inhibition of AA on sPPO and mPPO reached more than 95.42 ± 0.07% and 65.60 ± 1.16%, respectively. This indicates sPPO was more susceptible to AA compared with mPPO. The inhibitory effects of cinnamic acid followed the same trend of mPPO being relatively more stable than sPPO in the presence of cinnamic acid at these three concentrations. The effects of βCyD on inhibiting sPPO and mPPO were relatively weak even though its effects were increasing as the concentration was increased. Such a positive correlation between βCyD concentration and its inhibitory effect has also been found in potato PPO [20]. Cinnamic acid treatment can reduce the PPO activity in taro, and the inhibitory effect on taro PPO is also positively correlated with its concentration, which is consistent with the results of this experiment [31]. 

However, instead of a positive correlation between the inhibitor concentration and the inhibitory effect that was observed in the case of cinnamic acid, βCyD, and AA studies, the inhibitory effect of L-Cys on sPPO and mPPO activities apparently decreased as the concentration increased; at the same concentration, L-Cys showed a higher inhibitory effect on sPPO when compared with mPPO. It has been found that 0.83 mM L-Cys can effectively inhibit PPO-initiated browning in yam, resulting from L-Cys, as a thiol compound, reacting with quinones to form a stable colorless product; higher concentration of L-Cys (9.9 mM) almost reached 100% inhibition [13]. In this study, the highest inhibition on both forms of PPO by L-Cys was less than 35%. This shows that the same inhibitor can show different inhibitory effects on PPO from different sources; interestingly, differing from the inhibitory effect of L-Cys on sPPO, mPPO was even found to be significantly activated at the highest concentration (5 mM) of L-Cys. This was probably due to a conformational change of mPPO with higher concentrations of L-Cys. A similar activation phenomenon was found in the case of another sulfur-containing compound, sodium metabisulfite; mPPO activity in ‘Lijiang’ snow peach was significantly activated by 10 mM sodium metabisulfite, but with sPPO completely inhibited [30]. This indicates mPPO and sPPO may behave differently in the presence of sulfur-containing compounds.

Generally, from the data of Figure 1, AA exhibited the highest inhibitory effect on both sPPO and mPPO in peach fruits under current experimental conditions, indicating AA is still a desirable anti-browning agent for different forms of PPO. Compared with sPPO in peach fruit, mPPO was found to be more resistant to all inhibitors at all studied concentrations. Regarding the inhibition of different forms of PPO, the inhibition of several inhibitors on sPPO and mPPO activities of ‘Lijiang’ snow peach with catechol as substrate was reported by [30]. Their results showed that all inhibitors have a stronger inhibitory effect on sPPO than mPPO. This is comparable with the present data obtained in this study.

### 3.3. Effects of Inhibitors on sPPO and mPPO-Catalyzed Reactions with Different Substrates

**Figure 2:**
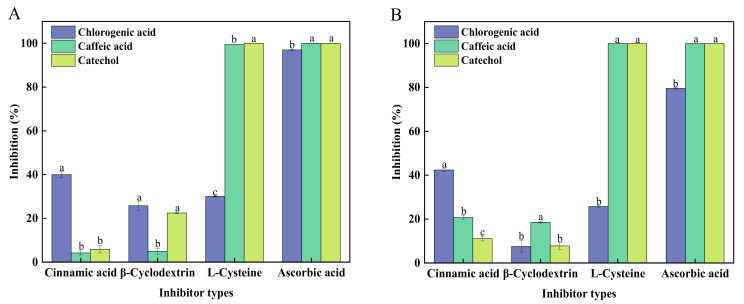
The effect of inhibitors on sPPO- (**A**) and mPPO- (**B**) catalyzed reactions with different substrates, including CA (final concentration of 3 mM), caffeic acid, and catechol (final concentration of 10 mM). The concentration of inhibitors studied in this experiment was 3 mM. Different letters indicate significantly different inhibitory effects when using different substrates (*p* < 0.05).

Peach fruit is rich in CA, caffeic acid, catechin, rutin, and other phenolic compounds that can be oxidized by PPO, and CA had been found to be one of the most important endogenous substrates for PPO in peaches [32,33]. Meanwhile, catechol is considered the standard substrate that has been widely used in most PPO studies [14,27]. Thus, three substrates (i.e., CA, caffeic acid, and catechol) were chosen to be studied in the present inhibition studies. Figure 2 compares the inhibitory effects of four different types of inhibitors on sPPO and mPPO activity when different substrates (i.e., CA, caffeic acid, and catechol) were used. The results show that with different substrates participating in PPO reactions, different inhibitors have different effects on sPPO and mPPO activities. When CA was used as the substrate, AA inhibited both sPPO and mPPO activities the most, and the inhibitory effect of cinnamic acid was significantly weaker, while L-Cys showed a much lower inhibitory effect, with βCyD exhibiting the lowest effect. When caffeic acid and catechol were used as substrates, both sPPO and mPPO activity were shown to be completely inhibited by AA and L-Cys. Their inhibitory effects were much higher compared with those obtained when CA was used as the substrate. It has been reported that 5 mM AA and 50 mM L-Cys can completely inhibit the activity of plum PPO on catechol [34]. 

When comparing inhibition of PPO reactions by a certain inhibitor with these three different substrates, cinnamic acid and βCyD were found not to inhibit the activities of sPPO and mPPO effectively. Cinnamic acid showed the highest inhibition percentage of both sPPO and mPPO activities on CA, but the percent inhibition was only approximately 40%. The effect of βCyD on mPPO inhibition was higher than that on sPPO with caffeic acid as the substrate, but sPPO was more inhibited during PPO-catalyzed CA or catechol oxidations. This indicates that sPPO and mPPO show different stabilities to βCyD in the presence of different substrates. 

PPO inhibitions with different substrates have also been investigated in other studies. It has been reported that the highest inhibition of PPO in gooseberry fruit was found when AA and quercetin were used as inhibitors, with 4-methylcatechol being the enzyme substrate; however, when using CA as the substrate, quercetin, L-Cys, and sodium sulfite showed stronger inhibitory effects on PPO [35]. This indicates that the different inhibitory effects of inhibitors were dependent on the studied substrates in PPO reactions.

### 3.4. Direct Effects of Inhibitors on sPPO and mPPO Activities

**Figure 3:**
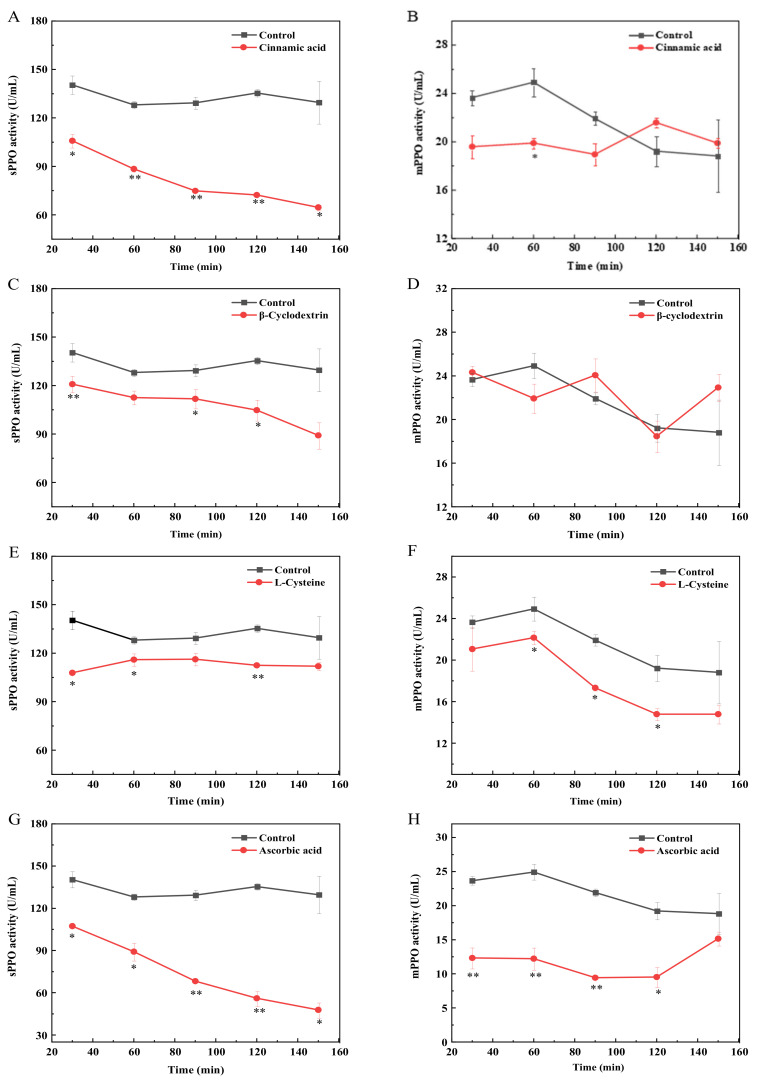
Effects of pre-incubation of sPPO and mPPO with inhibitors on subsequent PPO activity. Cinnamic acid with sPPO (**A**) and mPPO (**B**); βCyD with sPPO (**C**) and mPPO (**D**); L-Cys with sPPO (**E**) and mPPO (**F**); AA with sPPO (**G**) and mPPO (**H**). Final concentration of the four inhibitors was 3 mM. * indicates a significant difference between the groups with and without inhibitors at the same incubation time (*p* < 0.05); ** indicates a highly significant difference (*p* < 0.01).

Experiments discussed above mainly focused on investigating the PPO inhibition under different working conditions (i.e., inhibition with different concentrations of inhibitors, inhibition with different PPO substrates). To further determine the interactions between PPO and different inhibitors, direct effects of inhibitors on PPO activities (i.e., the influence of inhibitors on PPO in the absence of phenolic substrates) was investigated; specifically, PPO was incubated with inhibitors at 4 °C for 30–150 min prior to the determination of PPO activities. As shown in Figure 3, cinnamic acid, βCyD, L-Cys, and AA all had direct inhibitory effects on sPPO. When preincubating cinnamic acid and AA with sPPO, the subsequent sPPO activity decreased with the increase in incubation time. This indicates that the direct inhibitory effects of cinnamic acid, βCyD, and AA on inhibiting sPPO gradually increased as the incubation time increased. Compared with the inhibition of βCyD on sPPO, βCyD has no direct inhibitory effect on mPPO, while the other three inhibitors were found to directly inhibit mPPO; but when the incubation time was 150 min, the inhibitory effects of the four inhibitors on mPPO were not significant.

Among the four inhibitors, the effect of AA on PPO is the most widely studied [36,37]. With respect to its direct effect on PPO, it has been reported that AA can directly inhibit the activity of PPO from mushroom, causing an irreversible inactivation of PPO [38]. This is comparable with the results obtained in the present study, resulting from AA directly interacting with the active site of PPO. Additionally, for longer AA incubation with mPPO, AA can probably be gradually degraded due to its enzymatic or non-enzymatic oxidations to dehydroascorbic acid (DHA), resulting in no significant activity change when compared with that in the incubation mixture containing no AA [39]. For the direct inhibition of cinnamic acid on PPO, cinnamic acid contributes to a higher percent of direct inhibition on sPPO. This probably results from cinnamic acid more easily binding to the active site of sPPO compared with mPPO. A similar finding has been reported in taro PPO; at the protein level, cinnamic acid can also directly inhibit taro PPO activity by complexing copper at the active site of the enzyme [31]. 

Concerning the direct effect of βCyD on sPPO, it may be due to βCyD directly interacting with the functional groups in the active center of sPPO, leading to an irreversible direct inhibition. Contrary to the finding from Jiang et al., βCyD does not directly inactivate potato PPO [20]. This indicates that the direct effect on PPO shown by βCyD can be different because of the enzyme source. This experiment also showed a direct inhibitory effect of L-Cys on PPO, probably resulting from the SH groups in L-Cys binding to copper ions in the active center of PPO by replacing the histidine residue. Such a direct effect of L-Cys has also been found on PPO in fresh wet noodles [40]. But there are studies also showing that L-Cys reacts with bisphenol intermediates to form colorless compounds rather than directly inhibiting PPO in loquat [41]. In summary, the inhibitory effects on PPO from different sources are different and the two forms of PPO show different degrees of sensitivities to different inhibitors.

### 3.5. Comparison of Inhibition Types of sPPO and mPPO

**Figure 4:**
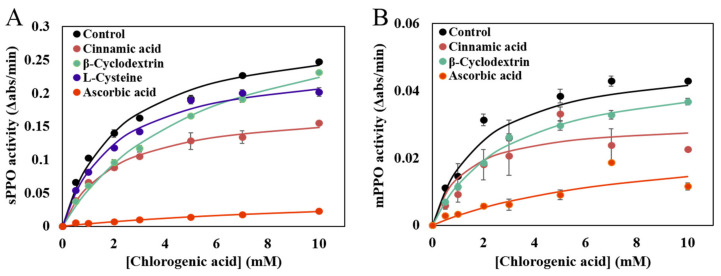
Substrates versus velocity plots based on peach (**A**) sPPO- and (**B**) mPPO-catalyzed CA oxidations in the presence and absence of different inhibitors.

Different concentrations of CA were used to obtain the kinetic parameters for PPO reactions with and without the four different inhibitors, as shown in Figure 4. According to the Michaelis– Menten equation, the Michaelis constant (K_m_) and the maximum velocity (V_max_) were estimated and compared. It can be seen from Table 2 that, in the absence of inhibitors, the Km values of sPPO and mPPO were basically the same, but the V_max_ of sPPO was much larger than that of mPPO. This indicates that both sPPO and mPPO show a similar affinity for the substrate CA. Based on the unchanged Km and decreased Vmax, cinnamic acid exhibited a non-competitive inhibitory effect on both sPPO and mPPO. This indicates that cinnamic acid may bind to the enzyme or the enzyme-substrate complex to finally form a complex of enzyme–substrate–inhibitor, which is catalytically inactive. Differently, cinnamic acid has showed a reversible mixed-type inhibitory effect on mushroom PPO [42]. L-Cys, like cinnamic acid, also shows a non-competitive inhibitory effect on sPPO. Such an effect of L-Cys on PPO has also been found in Whangkeumbae pear [43]. However, differing from the L-Cys effect on sPPO, the percent inhibition on mPPO was weakened and the activation effect was enhanced with the increase in CA concentration. Such an activation effect of mPPO by L-Cys is unclear and needs to be further investigated.

Kinetic parameters of PPO reactions in the presence of βCyD show that βCyD inhibition does not belong to the typical reversible inhibition types. βCyD has commonly been reported to be a cyclic compound with a relatively hydrophobic cavity and a hydrophilic periphery [20]. This property allows βCyD to accommodate the PPO substrate CA to form a βCyD-CA inclusion complex, resulting in a depletion of the substrate and decreased rate of PPO reaction. The substrate depletion is actually a special type of inhibition, and the enzyme kinetics shown based on such inhibition is very comparable with that shown in competitive inhibition. Thus, even though the kinetic parameters obtained did not show a substrate depletion exactly, the kinetic curve of the PPO reaction with βCyD herein showed a property comparable with that in a competitive inhibition, indicating βCyD inhibition of PPO is primarily due to the substrate depletion. Similar to βCyD, the kinetic parameters from the PPO reaction with AA did not show the characteristics of common reversible inhibitions. However, AA has been reported as a competitive inhibitor of lotus rhizome PPO that can compete with the substrate for binding to the active center of PPO [44]. Based on the present study, AA was found to directly inhibit both sPPO and mPPO (shown above). Also, with respect to the browning inhibition, it can reduce the quinone products generated from the PPO reactions back to their corresponding phenolic compounds, thereby preventing browning formation [43].

## 4. Conclusions

The present study mainly focused on determining the effects of four inhibitors on sPPO and mPPO in peach fruit. The results of this study showed that the specific activity of sPPO was higher than that of mPPO; this indicates sPPO was the main enzyme form that caused browning. Compared with mPPO, the four inhibitors all had more significant inhibitory effects on sPPO; while mPPO had higher resistance to the four inhibitors. Among several substrates studied, AA showed the highest inhibitory effect on both sPPO and mPPO compared with the other three inhibitors. L-Cys was found to activate mPPO at higher concentration when CA was used as the substrate; such an effect was not shown in the presence of the other two substrates since the activities of sPPO and mPPO with caffeic acid and catechol were completely inhibited by L-Cys. Also, with the exception of βCyD inhibition of mPPO, both sPPO and mPPO could be directly inhibited by the studied inhibitors. Cinnamic acid exhibited non-competitive inhibition on PPO under the conditions of PPO-catalyzed CA reaction, and L-Cys also showed the same inhibition on sPPO, while βCyD and AA inhibition of PPO did not belong to the typical reversible inhibition types. Further experiments related to the conformational change of PPO affected by different inhibitors need to be conducted. Overall, sPPO and mPPO behaved differently in the presence of different inhibitors and the inhibitory effect was highly associated with the source of PPO, the substrate environment, and the types and concentrations of inhibitors. This study provides a scientific basis for better prevention and control of peach browning during production, storage, and processing.

## Figures and Tables

**Table 1:** Partial purification of sPPO and mPPO from ‘HuJingMiLu’ peach fruit.

Purification Stages	Volume (mL)	Total Activity (U)	Protein Content (mg)	Specific Activity (U/mg)	Yield (%)	Purification Fold
sPPO						
Crude extract	264	2640	36	74	100	1
(NH4)_2_SO_4_ precipitation	28	912	10	95	35	1.3
mPPO						
Crude extract	20	179	7	24	100	1
(NH4)_2_SO_4_ precipitation	15	139	5	30	78	1.2

Note: One unit (U) of PPO activity was defined as the change in absorbance of 0.01 per minute. Protein content was estimated using the Bradford method.

**Table 2:** Comparison of kinetic parameters obtained from sPPO- and mPPO-catalyzed reactions.

Form	Inhibitors	K_m_ (mM)	V_max_ (Δabs/min)
sPPO	None	2.04 ± 0.23 ^c^	0.29 ± 0.01 ^b^
	Cinnamic acid	1.90 ± 0.10 ^c^	0.18 ± 0.00 ^d^
	β-Cyclodextrin	5.40 ± 0.31 ^b^	0.35 ± 0.00 ^a^
	L-Cysteine	2.12 ± 0.13 ^c^	0.25 ± 0.01 ^c^
	Ascorbic acid	11.18 ± 1.84 ^a^	0.05 ± 0.01 ^e^
mPPO	None	2.14 ± 0.30 ^b^	0.05 ± 0.00 ^a^
	Cinnamic acid	1.72 ± 0.76 ^b^	0.03 ± 0.01 ^b^
	β-Cyclodextrin	2.88 ± 0.07 ^b^	0.05 ± 0.00 ^a^
	L-Cysteine	**	**
	Ascorbic acid	5.99 ± 1.81 ^a^	0.02 ± 0.00 ^c^

Note: ** indicates that the kinetic parameters were not estimated as an activation of PPO was found in the presence of L-cysteine. Different lowercase letters represent significant differences in Km or Vmax between sPPO or mPPO groups with or without inhibitors (*p* < 0.05).

## Data Availability

Data are contained within the article.
